# Maternal Cypermethrin Exposure during the Perinatal Period Impairs Testicular Development in C57BL Male Offspring

**DOI:** 10.1371/journal.pone.0096781

**Published:** 2014-05-08

**Authors:** Chaobin Huang, Xiangdong Li

**Affiliations:** State Key Laboratory of Agrobiotechnology, College of Biological Sciences, China Agricultural University, Beijing, China; Ecole Normale Superieure de Lyon, France

## Abstract

Numerous studies have demonstrated that endocrine-disrupting compounds (EDC) are a possible cause of male reproductive organ malfunction and malformation. Cypermethrin (CYP) is a widely used synthetic pyrethroid and a potential EDC. This study aimed to examine the effects of perinatal exposure to low-dose CYP on the development and function of the offspring testes. Pregnant mice were intragastrically administered 0.12 to 12 mg/kg/day CYP from embryonic day 0.5 (E0.5) to weaning (PD21.5, postnatal day 21.5). Maternal exposure to 0.12, 1.2, and 12 mg/kg/day CYP affected the body and organ weight of the offspring. Exposure of CYP led to a dose-dependent decrease in the male-to-female sex ratio. A histopathological analysis revealed a thinner seminiferous epithelium layer at PD21.5, interstitial hyperplasia at PD45.5, and germ cell vacuolization at PD90.5 in the 12 mg/kg/day CYP group. The TUNEL assay results revealed increased germ cell apoptosis in the 12 mg/kg/day CYP group. The serum testosterone (T) level decreased, whereas the estradiol level increased with age in the 1.2 and 12 mg/kg/day CYP groups. The RT-PCR analysis demonstrated decreased expression of T production-related, mitosis-related, and meiosis-related genes in the 1.2 and 12 mg/kg/day CYP groups. The *in vitro* experimental results demonstrated reduced expression of steroidogenesis genes and decreased T levels. It is concluded that perinatal exposure to low-dose CYP affects testes development and function in adults.

## Introduction

Compounds that can mimic and block natural hormones and cause adverse health effects in humans and wildlife are referred to as endocrine-disrupting compounds (EDCs) [1], [2]. Studies have demonstrated that a wide range of EDCs can lead to serious problems, such as infertility [3]–[7].

Cypermethrin (CYP), a type II synthetic pyrethroid insecticide, replaces traditional organochlorine and organophosphate pesticides and has been widely used [8]. Different studies had indicated that CYP treatment decreases the layers of spermatogenic cells, increases the inside diameter of seminiferous tubules, decreases Star expression in adolescent mice [9], disturbs the array of spermatogenic cells [10], reduces sperm count and motility in male mice [7], [11]–[14], decreases serum testosterone (T) levels, and increases serum follicle-stimulating hormone (FSH) and luteinizing hormone (LH) levels [15]. It has been demonstrated that CYP exerts anti-androgen effects in androgen receptor reporter gene assays [16], [17] and can induce ER transactivity [3]. Most studies have used higher doses of CYP, ranging from 39.66 mg/kg/day [7] to 485 mg/kg/day [12] and even toxicological doses, and most studies have focused on postnatal exposure [9], [10], [12], [15], [18]. However, there are no reports describing the effects of lower dosage or environmental exposure levels during fetal exposure on the growth and development of testes. Various studies have investigated the effects of EDCs on the growth and development of the fetus, which is sensitive to hormonal fluctuations [19]–[22]. Impaired reproductive development has been demonstrated in the sons of female gardeners or farmers where pesticides have been used [23]–[28]. This study aimed to assess CYP exposure during the perinatal period to determine its effect on fetal development and its long-term impact on male reproduction in C57BL mice.

## Materials and Methods

### *In vivo* experimental design, treatment, and sample collection

It has been reported that the LD50 of CYP in mice is 250.0 mg/kg when administered orally in corn oil [15]. Some toxicological studies have used a dosage equal to 1/5LD50 or higher [12], [15]. It has been reported that the environmental residue of CYP in surface water varies in different countries from 0.022 to 5.6 ppb [29]–[32], whereas the CYP residue in human milk can be as high as 945.1 to 1443.8 ppb [33]. Based on these studies, we chose CYP exposure doses of 0.12, 1.2, and 12.0 mg/kg/day for the *in vivo* experiment. Gestating C57BL/6 mice were treated with different doses of CYP through intragastric administration from embryonic day 0.5 (E0.5, day of plug) to weaning (postnatal day 21.5, PD21.5). The gestating control group was treated with vehicle (corn oil; n = 8–10 female mice/group). After birth, sex determination was conducted through *sry* amplification [34] (Fig. S1), and the male offspring were subjected to further analyses. The body weights of the male offspring were monitored every five days. The male mice were anaesthetized with pentobarbital sodium (40 mg/kg) and sacrificed randomly by cardiac puncture at three time points: PD21.5 (weaning day, n = 3–10), PD45.5 (juvenile phase, n = 5–10), and PD90.5 (maturation phase, n = 5–11). Serum samples were stored at −20°C until hormone measurement. The testes were dissected and separated into two parts. One part was fixed with 4% paraformaldehyde (PFA) or Bouin's solution (Sigma, USA) for testicular immunohistochemical, apoptosis, and pathological analyses. The other part was snap-frozen in liquid nitrogen and stored at −80°C until use. The testes, epididymises, and livers were weighed.

### Sex determination of offspring

The expression of the *Sry* gene [35] was detected through the amplification of tail DNA. The extracted DNA was denatured at 95°C for 5 min and amplified through 30 cycles of PCR using the following conditions: 95°C for 30 sec, 55°C for 30 sec, and 72°C for 30 sec. The PCR product was subjected to agarose gel electrophoresis and was visualized by ethidium bromide staining. *L19* was amplified as an internal control.

### Ethics statements

The Ethics Committee for Animal Experimentation of the China Agricultural University approved all of the animal experiments (Register No. SKLAB-2011-01-03).

### CYP detection

The CYP levels of the prepared dosages and serum were measured by ELISA following the manufacturer's protocol (R&D Systems, USA). Briefly, after proper dilution, the standards and samples were added to a 96-well plate (coated with CYP-coupling antigen). The antibody-enzyme conjugate was then added, and the plate was incubated for 30 min at 37°C and washed. The color-reagents A and B were then added to each of the wells and the plate was incubated for 10 min at 37°C. The stop solution was then added, and the absorbance value at 420 nm was read. The standard curve was generated according to the standard values to calculate the concentration of each of the samples.

### Morphological and histological analyses

The testes were embedded in paraffin, and sections (5-µm thick) were deparaffinized using xylene and then stained with hematoxylin and eosin (HE) through standard procedures for histopathological examination. The mounted slides were examined with a light microscope (IX71, Olympus, Japan), and photos were taken with a Nikon DXM1200F camera (Nikon, Japan). The quantification of germ cell numbers and germ cell vacuolation was conducted by counting 5–20 related slides.

### Immunohistochemical analyses

The immunohistochemical analyses were performed as previously described [36]. The slides were stained with the following antibodies for chromatic visualization: goat anti-3β-HSD, rabbit anti-CYP17A1 (further information on the primary antibodies is presented in Table 1, purchased from Santa Cruz, USA), biotinylated rabbit-anti-goat, goat-anti-rabbit IgG (all at 1∶200 dilution, Vector Laboratories, USA), and streptavidin-conjugated HRP (1∶200 dilution, Jackson ImmunoResearch, USA). The specific binding was visualized using DAB (1∶20 dilution, Leica, Germany). The sections were counterstained with hematoxylin and mounted for further microscopic analyses. The numbers of positive cells in the testes at PD21.5 and the testes at PD45.5 and PD90.5 were counted in five randomly and ten randomly selected fields, respectively. A total of 5-20 related slides were used to quantify the number of positive cells.

**Table 1:** Antibodies information.

Antibody	Dilution	Cat No.	Source
β-Actin	1∶500^1^	sc-47778	mouse monoclonal IgG
Star	1∶500^1^	sc-25806	rabbit polyclonal IgG
3β-HSD	1∶200^2^ or 1∶500^1^	sc-30820	goat polyclonal IgG
Cyp17a1	1∶500^1^	sc-66850	rabbit polyclonal IgG
17β-HSD3	1∶500^1^	sc-66415	goat polyclonal IgG
Pcna	1∶200^2^ or 1∶500^1^	sc-7907	rabbit polyclonal IgG
AR	1∶500^1^	sc-816	rabbit polyclonal IgG
ERα	1∶500^1^	sc-542	rabbit polyclonal IgG

1 represents dilution used in Western blot assay,

2 represents dilution used in immunohistochemical assay.

### Terminal dUTP nick-end labeling staining

The paraffin-embedded slides were stained with the terminal dUTP nick-end labeling (TUNEL) technique to detect the apoptosis of germ cells. An *in situ* cell death detection kit (AP, Roche, Switzerland) was applied according to the manufacturer's protocols. The number of TUNEL-positive cells was counted through immunohistochemical analyses. The quantification of the positive cell number was conducted by counting 5-20 related slides.

### RNA extraction and semi-quantitative RT-PCR

The total RNA from the testes and mLTC-1 cells (CRL-2065, ATCC, USA) was isolated using the TRIzol reagent (Invitrogen, USA). In addition, pre-PCR was performed to synthesize the cDNA for the semi-quantitative RT-PCR analyses. One microgram of total RNA was incubated with 10 U of avian myeloblastosis virus reverse transcriptase (Promega, USA), dNTP mix, and oligo-dT primers at 42°C for 1 h. The cDNAs were then denatured at 95°C for 5 min and amplified through 20–36 cycles of PCR using the following conditions: 95°C for 30 sec, 51.5°C to 61°C for 30 sec, and 72°C for 30 sec. An aliquot of the RT-PCR product was subjected to agarose gel electrophoresis and visualized by ethidium bromide staining. The density of the gel bands was quantified using the ImageJ software, version 1.34 (NIH). L19 was amplified as an internal control of the total amount of RNA used. The primer sets are given in Table 2.

**Table 2:** The sequences of primers, annealing temperature and amplified products of PCR.

Gene name	Primer	Annealing (°C)	Product size (bp)
*L19*	F: 5′-gaaatcgccaatgccaact-3′	56	406
	R: 5′-tgagactcgcaggtctaaga-3′		
*Star*	F: 5′-agatgtgggcaaggtgtttc-3′	56	387
	R: 5′-gagcagccaagtgagtttagt-3′		
*Cyp11a1*	F: 5′-gctgcctgggatgtgattt-3′	53.5	548
	R: 5′-cggaagtgggtggtatttt-3′		
*3β-HSD*	F: 5′-aatctgaaaggtacccagaa-3′	51.5	360
	R: 5′-tcatcatagctttggtgagg-3′		
*Cyp17a1*	F: 5′-ccaggacccaagtgtgttct-3′	56	250
	R: 5′-cctgatacgaagcacttctcg-3′		
*17β-HSD3*	F: 5′-attttaccagagaagacatct-3′	52	367
	R: 5′-ggggtcagcacctgaataatg-3′		
*Cyp19a1*	F: 5′-gcttctcatcgcagagtatccg-3′	60	266
	R: 5′-caagggtaaattcattgggctg-3′		
*AR*	F: 5′-ctgggaagggtctaccac-3′	55	128
	R: 5′-ggtgctatgttagcggcctc-3′		
*ERα*	F: 5′-cgtgtgcaatgactatgcctc-3′	55	199
	R: 5′-tttcatcatgcccacttcgtaa-3′		
*Pcna*	F: 5′-caacttggaatcccagaac-3′	53	294
	R: 5′-agacagtggagtggctttt-3′		
*Nanos3*	F: 5′-tgcaggcaaaaagctgacc-3′	60	101
	R: 5′-cttcctgccacttttggaac-3′		
*Cyclin D2*	F: 5′-cgatgattgcaactggaagc-3′	54	168
	R: 5′-ttcagcagcagagcttcgat-3′		
*Stra8*	F: 5′-gtttcctgcgtgttccacaag-3′	54	150
	R: 5′-cacccgaggctcaagcttc-3′		
*Cyclin A1*	F: 5′-gatgtgtatgaagtcgacacc-3′	54	91
	R: 5′-gtggggtcaaccagcattgg-3′		
*Sry*	F: 5′-tcttaaactctgaagaagagac-3′	55	404
	R: 5′-gtcttgcctgtatgtgatgg-3′		
*Insl3*	R: 5′- ggagccgaagtcgagactg-3′	55	186
	R: 5′- gccatctagtccacccctc-3′		

F, forward; R, reverse.

### Western blot analyses

The Western blots were performed as described previously [36]. Briefly, the total protein from the cells was extracted using RIPA buffer. Aliquots of protein were electrophoresed in SDS-PAGE and transferred to a PVDF membrane. The membrane was incubated with mouse anti-β-Actin, rabbit anti-Star, goat anti-3β-HSD, rabbit anti-CYP17A1, goat anti-17β-HSD3, rabbit anti-Pcna, rabbit anti-AR, and rabbit anti-ERα (further information on the primary antibodies are shown in Table 1, purchased from Santa Cruz, USA). After washing with TBST buffer, the membrane was then incubated with HRP-labeled goat-anti-mouse IgG, goat-anti-rabbit, and rabbit-anti-goat IgG (all at 1∶2000 dilution, Jackson ImmunoResearch, USA). The final exposure was obtained using enzymatic chemiluminescence (GE Healthcare, USA). The film was then scanned, and the band density was quantified using the ImageJ software, version 1.34 (NIH).

### Cell culture and treatment

It has been reported that the pyrethroid metabolite concentration in human urine ranges from 0.318 to 189 ppb [37]–[41] and that the CYP residue in human serum can be as high as 0.3 ppm [42]. We chose to use a CYP concentration of 1×10^−7^−1×10^−5^ M (0.42–42 ppb) in the medium. Leydig mLTC-1 cells were cultured in RPMI1640 containing 100 U/ml penicillin and 100 µg/ml streptomycin at 37°C under a 5% CO_2_ atmosphere. Serum starvation was performed for 24 h before treatment, and the cells were then treated with three doses of CYP (10^−7^ M, 10^−6^ M, and 10^−5^ M) at 70% confluence for 24 h. DMSO (1/10^7^) was used as the vehicle control, and LH (5 IU/ml) was used as a positive control. The total RNA and protein were extracted for further analyses.

### Measurement of hormone levels

The T and E_2_ levels in the serum or culture medium were measured by radioimmunoassay after diethyl ether extraction following the manufacturer's protocol, as described previously [36].

### Statistical analyses

The Data were analyzed for statistical significance using SPSS 12.0.1 (SPSS, USA). The data for all of the groups were first tested for normality through the Shapiro-Wilk test. If normally distributed, they were then compared using one-way ANOVA to determine the differences between the treated groups and the vehicle group. Pearson's correlation analysis was performed to determine the dose-response relationship. P values less than 0.05 were set as statistically significant. All of the values are presented as the means ± SEM (standard error of mean). All of the graphs were generated with GraphPad Prism 5.0 (GraphPad, USA).

## Results

The serum CYP residues of mothers and F1 male mice were determined using the ELISA method (Fig. S2). The concentrations ranged from 5.04 to 169.84 ppb in the mothers and from 0.57 to 7.63 ppb in the F1 male mice. We speculated that CYP can be transmitted from the mother to the offspring through blood and (or) milk. There was a nonsignificant difference between the treated groups and the control group in overall body weight (BW) (PD0.5–PD90.5) with no observed abnormalities in the liver histology among the groups. At PD21.5, the exposure of pregnant mice to 0.12, 1.2, and 12 mg/kg/day CYP significantly decreased the BW, testis weight (TW), and liver weight (LW) in the male offspring. At PD45.5, the TWs of the two treated groups were increased compared with that of the control group. At P90.5, the BWs of the treated groups were increased. There were nonsignificant differences in the epididymis weight among all of the groups (Table 3). Interestingly, the male-to-female ratio decreased in a dose-dependent manner (Table 4). In addition, lethal embryos were observed at E14.5 of pregnancy, and there were more sites of lethal embryos in the CYP group than in the control group (Fig. S3).

**Table 3:** Offspring body and organs weight.

Group	Body weight (g)			Liver weight (g)		
	PD21.5	PD45.5	PD90.5	PD21.5	PD45.5	PD90.5
Con	13.65±0.97^a^	32.74±1.30	38.21±2.57^a^	0.67±0.06^a^	2.07±0.11	2.11±0.20
0.12 mg/kg/day	11.09±0.29^b^	33.95±0.76	39.76±0.81^b^	0.43±0.12^b^	2.10±0.21	2.22±0.18
1.2 mg/kg/day	9.31±0.97^b^	34.42±1.30	41.10±1.13^b^	0.38±0.05^b^	2.02±0.06	2.14±0.09
12 mg/kg/day	10.35±0.72^b^	34.82±0.63	44.17±0.73^b^	0.36±0.04^b^	2.08±0.09	2.37±0.07

Group	Testis weight (g)			Epididymis weight (g)		
	PD21.5	PD45.5	PD90.5	PD21.5	PD45.5	PD90.5
Con	0.08±0.00^a^	0.17±0.04^a^	0.29±0.01	0.01±0.00	0.06±0.00	0.09±0.00
0.12 mg/kg/day	0.06±0.01^b^	0.20±0.02^b^	0.28±0.03	0.01±0.00	0.07±0.01	0.09±0.01
1.2 mg/kg/day	0.04±0.01^b^	0.22±0.01^b^	0.27±0.02	0.01±0.00	0.06±0.00	0.09±0.00
12 mg/kg/day	0.04±0.01^b^	0.23±0.01^b^	0.28±0.01	0.01±0.00	0.07±0.00	0.10±0.00

Con, control group. Different superscripts (^a^, ^b^) depict significant differences among body or organs weight (*P*<0.05).

**Table 4:** Number of offspring and sex ratio of male to female.

Group	Number of offspring			Male to female ratio
	Male	Female	Total	
Con	31	30	61	1.03:1
0.12 mg/kg/day	13	15	28	0.87:1
1.2 mg/kg/day	29	38	67	0.76:1
12 mg/kg/day	22	32	54	0.69:1

**Figure 1:**
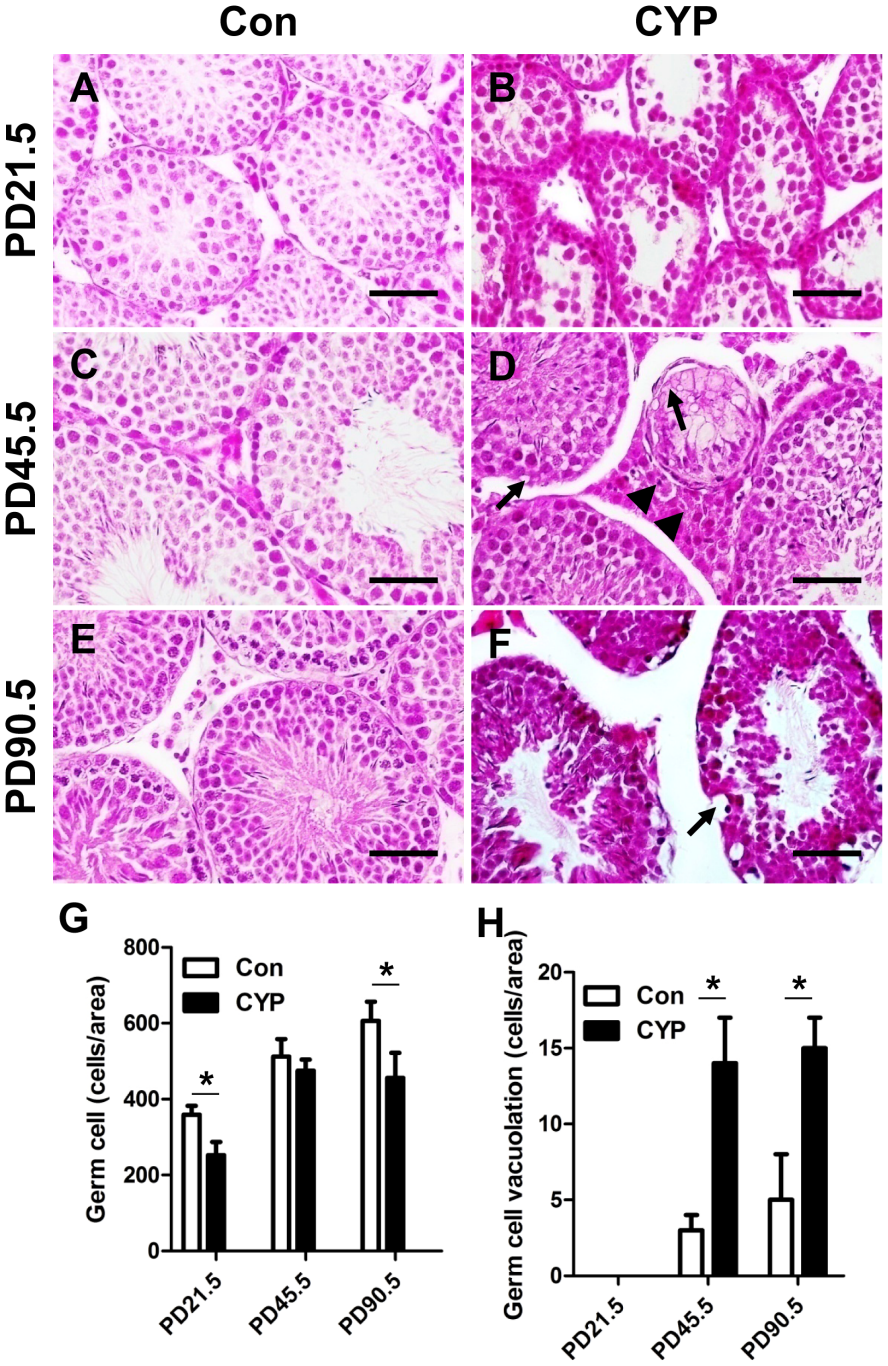
Effects of maternal perinatal CYP-exposure on the testicular histology of offspring. (A) Testicular histology of the control group offspring at PD21.5. (B) Testicular histology of the 12 mg/kg/day CYP-treated group offspring at PD21.5: the seminiferous epithelium layer was thinner than that of the control. (C) Testicular histology of the control group offspring at PD45.5: normal spermatogenesis was observed. (D) Testicular histology of the 12 mg/kg/day CYP-treated group offspring at PD45.5. Some of the germ cells displayed vacuolation (arrow) and hyperplasia of interstitial cells (arrowhead). (E) Testicular histology of the control group offspring at PD90.5. The lumen of the seminiferous tubules was filled with spermatozoa. (F) Testicular histology of the 12 mg/kg/day CYP-treated group offspring at PD90.5. More severe vacuolation of germ cells and destruction of the seminiferous epithelium were observed. All of the images were taken at 400× magnification. Scale bars, 40 µm. (G) Quantification of germ cells in 5–20 related slides of A–F. (H) Quantification of germ cell vacuolation in 5–20 related slides of A–F. The data represent the mean ± SEM. *P<0.05.

The histological analysis indicated that the seminiferous epithelium of the CYP-treated testes appeared thinner and that the germ cell number was decreased compared with the vehicle-treated group at PD21.5 and PD90.5 (Figs. 1A/B, and E–G). The number of Leydig cells was increased at PD45.5 in the CYP group (Figs. 1C/D). The number of germ cell vacuolations in the CYP group was markedly higher at PD45.5 and PD90.5 than in the control group (Figs. 1C–F and H).

**Figure 2:**
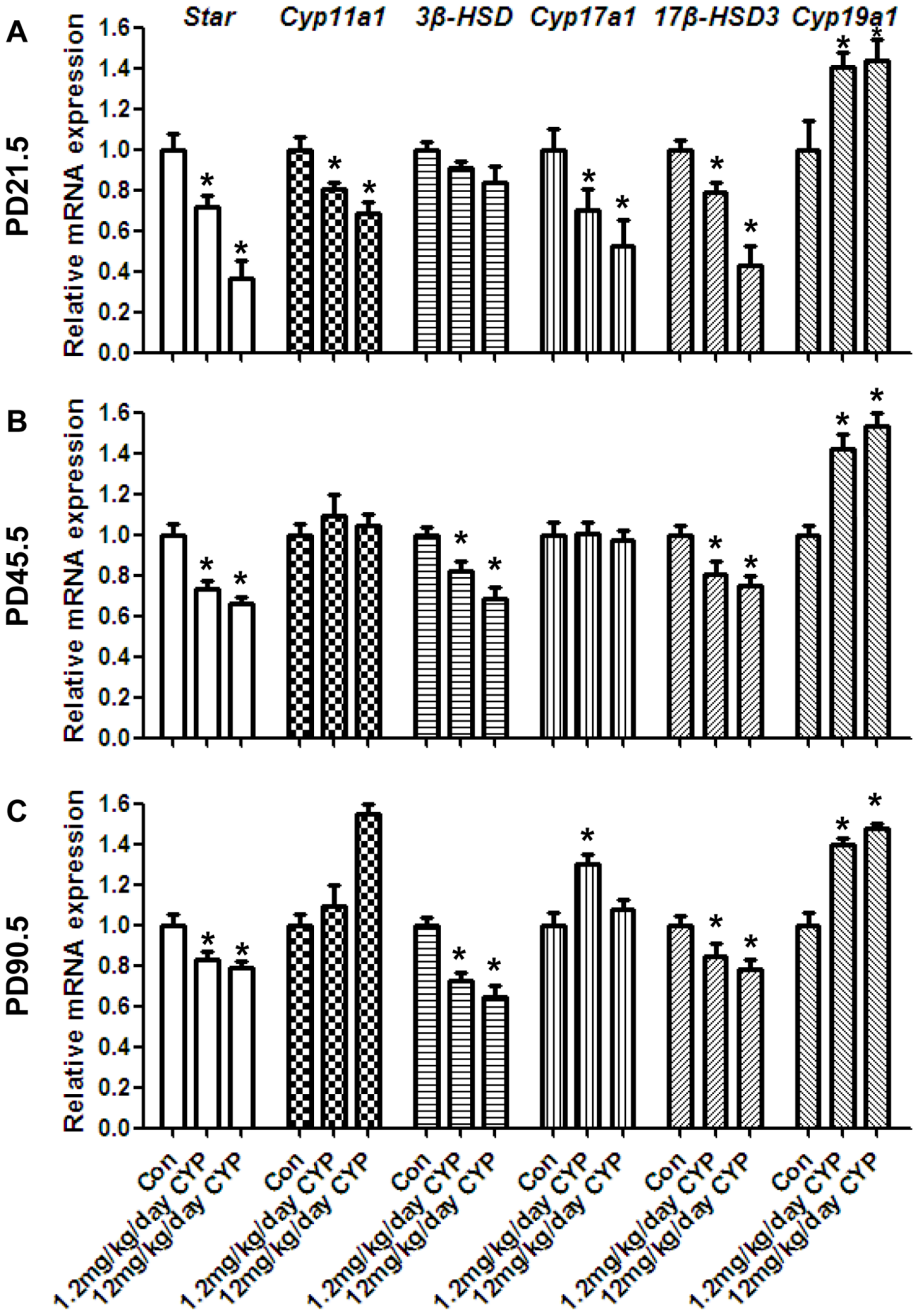
Effects of maternal perinatal CYP-exposure on testicular steroidogenesis-related genes in offspring. (A) The mRNA levels of *Star*, *Cyp11a1*, *3β-HSD*, *Cyp17a1*, *17β-HSD3*, and *Cyp19a1* in the testes at PD21.5 using RT-PCR. *Star*, *Cyp17a1*, and *17β-HSD3* were significantly downregulated in the CYP exposure groups. *Cyp11a1* displayed a downward trend, but this trend was not significant. However, *Cyp19a1* was upregulatedand, and there was no change in *3β-HSD*. (B) mRNA levels of steroidogenesis-related genes at PD45.5. *Star*, *3β-HSD*, and *17β-HSD3* were significantly downregulated in the CYP exposure groups. *Cyp19a1* was upregulated, and there were no changes in the *Cyp11a1* and *Cyp17a1* expression levels. (C) mRNA levels of steroidogenesis-related genes at PD90.5. *Star*, *3β-HSD*, and *17β-HSD3* were downregulated significantly in the CYP exposure groups, and *Cyp19a1* was upregulated significantly. The data represent the mean ± SEM. *P<0.05.

**Figure 3:**
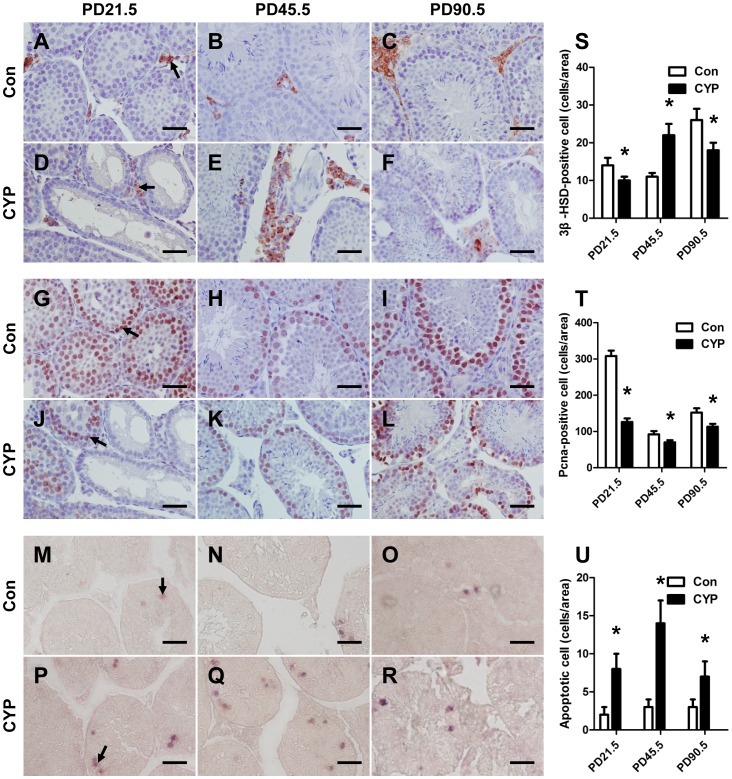
3β-HSD, Pcna immunohistochemistry and TUNEL assay of testes. Slides of (A, B, C) control and (D, E, F) CYP-treated offspring at PD21.5, PD45.5, and PD90.5, respectively, were immunolocalized with antibody for 3β-HSD. Slides of (G, H, I) control and (J, K, L) CYP-treated offspring at PD21.5, PD45.5, and PD90.5, respectively, were immunolocalized with antibody for Pcna. Slides of (M, N, O) control and (P, Q, R) CYP-treated offspring at PD21.5, PD45.5, and PD90.5, respectively were stained using the TUNEL method. All of the images were taken at 400× magnification. Scale bars, 40 µm. (S) Quantification of 3β-HSD-positive Leydig cells in 5–20 related slides of A–F. (T) Quantification of Pcna-positive germ cells in 5–20 related slides of G–L. (U) Quantification of apoptotic germ cells in 5–20 related slides of M–R. The data represent the mean ± SEM. *P<0.05.

**Figure 4:**
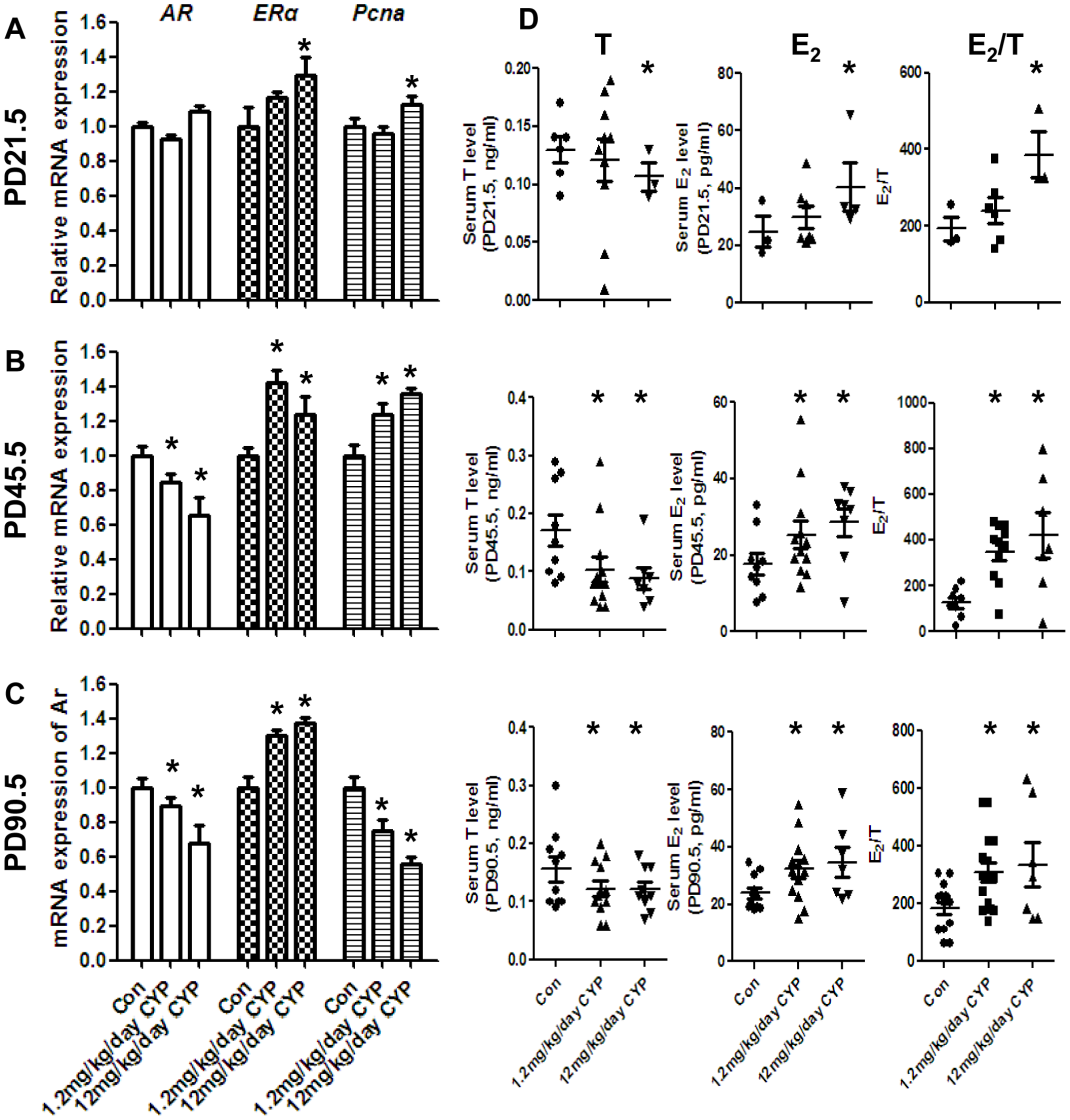
Effects of maternal CYP-exposure on the AR, ERα, Pcna, and E2/T levels of offspring. (A) The mRNA level of *AR* was not affected at PD21.5. The *ERα* level was upregulated significantly in the 12 mg/kg/day-exposure group, and there was an increasing trend in the other exposure group. *Pcna* expression was not altered. (B) *AR* expression was downregulated significantly in the exposure groups, and *ERα* and *Pcna* were upregulated, which may explain the hyperplasia of interstitial cells. (C) *AR* and *Pcna* were downregulated significantly, and *ERα* was upregulated, as observed at PD21.5 and PD45.5. (D) Serum T and E_2_ levels and the E_2_/T ratio at the three time points. The T level was lower, and the E_2_ level was higher in the CYP exposure groups at PD45.5 and PD90.5. The data represent the mean ± SEM. *P<0.05.

We then examined the steroidogenesis of all of the groups. The expressions levels of these genes were increased with increased age (Fig. S4). The analysis of the different treatment groups revealed that *Star* expression was decreased significantly at the three time points in the CYP groups, and the same results were observed in the analysis of the expression of *3β-HSD* and *17β-HSD3* (Fig. 2). The expression of *Cyp11a1* and *Cyp17a1* was decreased at PD21.5, whereas *Cyp19a1* was upregulated at the three time points in the CYP groups. The immunohistological results indicated that the number of 3β-HSD-positive cells in the CYP group was lower than that observed in the vehicle group at PD21.5 and PD90.5, but the number was higher at PD45.5 (Figs. 3A–F, 3S). Th serum T and estradiol (E_2_) levels (Fig. 4D) verified the RT-PCR results, and the T levels of the two CYP-treated groups were lower than that of the vehicle group (Fig. 4D left), whereas the E_2_ level was higher in the CYP groups at PD45.5 and PD90.5 (Fig. 4D right). *AR* was downregulated at PD45.5 and PD90.5 (Figs. 4A–C). In contrast, *ERα* was upregulated at all three time points (Figs. 4A–C). *Pcna* was upregulated at PD45.5 but downregulated at PD90.5 in the CYP groups (Figs. 4A–C). The same results were found with the immunohistological analysis (Figs. 3G–L, 3T). *Insl3*, an important regulatory factor in testicular descent and germ cell apoptosis [43], was also decreased in the CYP groups (Fig. S5).

**Figure 5:**
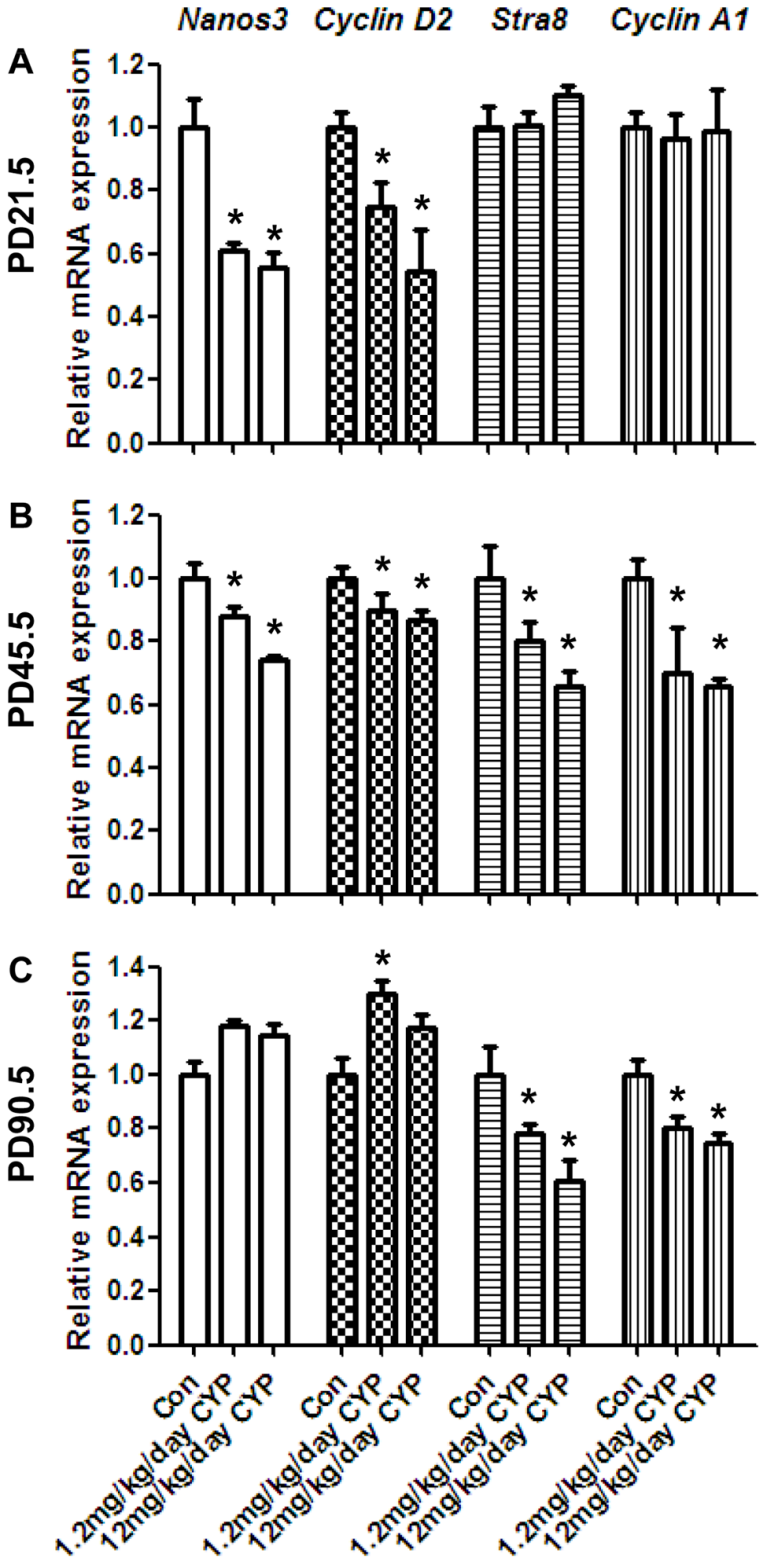
Effects of maternal perinatal CYP-exposure on mitosis and meiosis markers. (A, B, C) mRNA levels of the mitosis markers *Nanos3* and *Cyclin D2* and the meiosis markers *Stra8* and *Cyclin A1* at PD21.5, PD45.5, and PD90.5. The mitosis markers in the exposure groups were downregulated at PD21.5. The mitosis markers were also downregulated at PD45.5. At PD90.5, the mitosis markers were increased, but not significantly, and the meiosis markers were downregulated, as was observed at PD45.5. The data represent the mean ± SEM. *P<0.05.

Because of the altered germ cell number, we investigated the gene expression level of mitosis and meiosis markers. At PD21.5, *Nanos3* and *Cyclin D2* were decreased in the CYP groups (Fig. 5A). At PD45.5, the *Nanos3*, *Stra8*, and *Cyclin A1* mRNA levels were decreased (Fig. 5B). At PD90.5, *Stra8* and *Cyclin A1* were significantly decreased in the CYP groups (Fig. 5C). The number of Pcna-positive proliferating germ cells was decreased in the CYP groups at the three time points (Figs. 3G–L, 3T), whereas the TUNEL assay results indicated increased number of apoptotic germ cells in the CYP group at the three time points (Figs. 3M–R, 3U).

**Figure 6:**
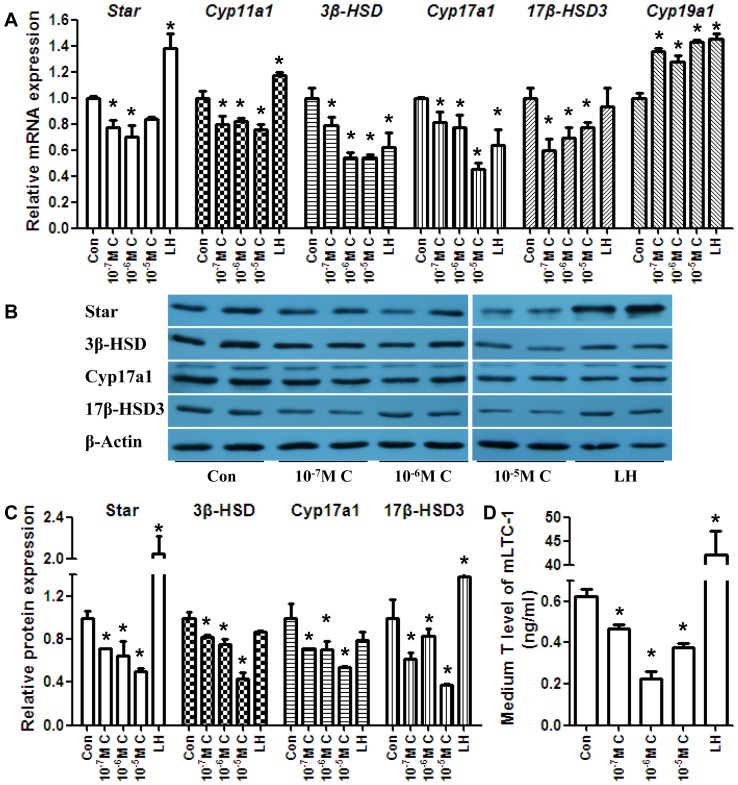
Effects of CYP-treatment on steroidogenesis-related genes and media T levels in mLTC-1 cells. (A) The mRNA levels of mLTC-1 steroidogenesis-related genes. (B) The protein levels of mLTC-1 steroidogenesis-related genes. (C) Quantitative analysis of scanning densitometry of protein levels of mLTC-1 steroidogenesis-related genes from (B). (D) Media T levels of mLTC-1 cells. The mRNA and protein levels of Star, 3β-HSD, Cyp17a1, and 17β-HSD3 were downregulated significantly compared with the control group. The mRNA and protein levels of Cyp19a1 were upregulated after CYP treatment. The media T levels were downregulated. LH was used as a positive control. The data represent the mean ± SEM. *P<0.05. C, CYP.

**Figure 7:**
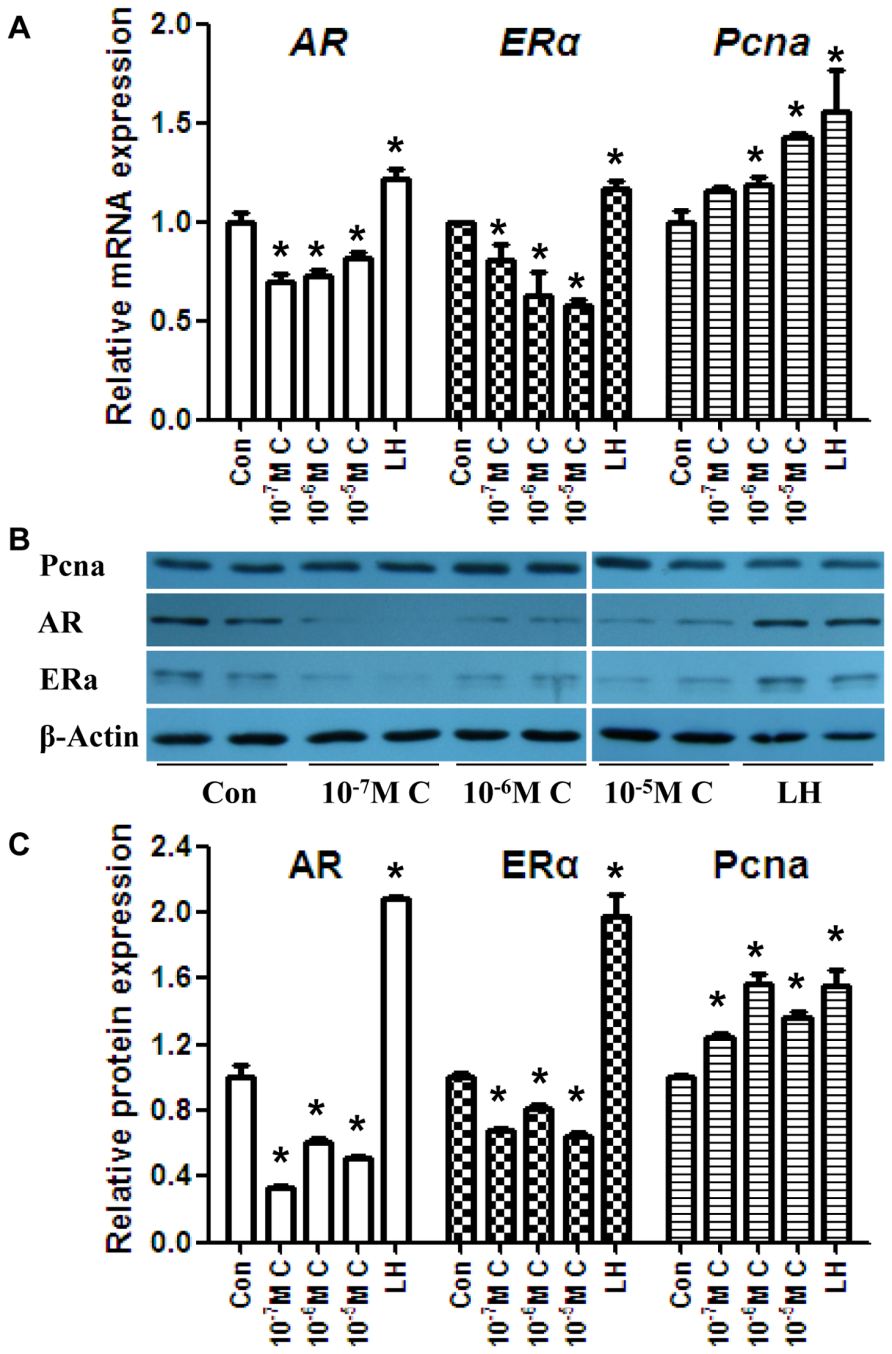
Effects of CYP-treatment on AR, Erα, and Pcna in mLTC-1cells. (A) The mRNA levels of *AR*, *ERα*, and *Pcna* in mLTC-1 cells. (B) The protein levels of AR, Erα, and Pcna in mLTC-1 cells. (C) Quantitative analysis of scanning densitometry of protein levels from (B). The mRNA and protein levels of AR were downregulated after CYP treatment, which is consistent with the T levels. In contrast, the ERα mRNA and protein levels were upregulated. The Pcna mRNA and protein levels suggest that CYP treatment may promote the proliferation of Leydig cells. The data represent the mean ± SEM. *P<0.05. C, CYP.

The *in vivo* results indicated the disruption of steroidogenesis after maternal exposure. We treated the mLTC-1 Leydig cell line with CYP (LH was used as a positive control) to determine the effect of CYP on the basal expression level of related genes. LH treatment increased the *Star* mRNA expression level. CYP treatment decreased *Star, Cyp11a1*, *3β-HSD*, *Cyp17a1*, and *17β-HSD3* expression, whereas *Cyp19a1* was increased. The Star protein level was elevated significantly post-LH induction. The protein levels of Star, 3β-HSD, Cyp17a1, and 17β-HSD3 were decreased post-CYP treatment (Figs. 6B/C). CYP treatment led to decreased T production (Fig. 6D). The expression of AR was downregulated in the CYP group. In contrast, the expression of ERα was downregulated, and the expression of Pcna was upregulated at both the mRNA and protein levels (Figs. 7A–C). Another set of *in vitro* experiments was also conducted, and these included a vehicle control group, a vehicle+LH group, and a CYP+LH group (Fig. S6).

## Discussion

Most studies have reported that direct exposure to high-dose of CYP impairs male reproduction [10], [18], [44]. We investigated the low-dose effects of CYP exposure during the perinatal stage and found impaired testicular development and steroidogenesis in the male offspring. The male-o-female ratio, BW, and TW were decreased at PD21.5. The structure of the seminiferous epithelium layer was also changed. The expression levels of steroidogenesis genes and hormone receptors were altered after CYP exposure both *in vivo* and *in vitro*. The mitosis and meiosis markers were also changed. The serum T levels were decreased, and E_2_ levels were increased.

Androgen is a prerequisite for normal spermatogenesis and development [45], and the binding of androgen to the AR plays an important role in the induction of the male external genitalia during embryonic differentiation and spermatogenesis [46]. In fetal and neonatal testes, AR expression is restricted to the interstitial compartment [47], [48]. Merlet *et al.* observed testicular dysgenesis during the embryonic period of gender differentiation in AR knockout mice [49]. Thus, perinatal CYP exposure may affect the precursors of adult Leydig cells. *Star* and *3β-HSD* were downregulated significantly in the CYP groups; in addition, in these groups, the T level was reduced, and the *Cyp19a1* and E_2_ levels were increased. Moreover, the AR hormone receptor was downregulated, and ERα was upregulated. An imbalance of androgenic and estrogenic signals may lead to serious structural abnormalities. Previous results have demonstrated that mice overexpressing human aromatase possess a multitude of structural and functional alterations in the reproductive organs [50], [51], and a decreased male-to-female ratio may arise from this overexpression. Taken together, the current results indicate that the inhibition of the androgenic signal during the prenatal and neonatal periods impairs the ability of Leydig cells to produce T in favor of E_2_ due to the overexpression of aromatase.

The apoptosis of spermatogonia and spermatocytes occurs in the mitotic phase [52], [53]. Studies have also found that deltamethrin and diethylstilboestrol induce a greater degree of apoptosis in adult male testes [54], [55]. In this study, we found much greater apoptosis of germ cells in the CYP groups. Sufficient T plays a vital role in the inhibition of germ cell apoptosis [56]. Reduced T levels lead to the separation of germ cells from the epithelium of the seminiferous tubules [57]. In the present study, we found that the serum T levels were decreased significantly by maternal CYP exposure, which will weaken their ability to maintain spermatogenesis. Studies on bisphenol A [58], [59] and hexachlorocyclohexane [60] have demonstrated that EDCs can affect mitosis and meiosis, and we also found that the expression of mitosis and meiosis marker genes was altered. The levels of Nanos3, which is important for maintaining undifferentiated spermatogonia [61], and the cell cycle regulator Cyclin D2 [62] were evaluated. We found that the expression of these two genes was decreased in the CYP groups at PD21.5 and PD45.5. The analysis of Stra8, which regulates meiotic initiation in spermatogenesis [63]–[65], and Cyclin A1, which is a meiosis-specific cyclin [66], [67], revealed that these genes were decreased at PD45.5 and PD90.5. Our results illustrated that spermatogenesis and steroidogenesis were affected by CYP treatment and that the vacuolation of germ cells may be a result of the decreased expression of these key proteins and the reduced T levels.

The male-to-female ratio at birth is a marker of parental endocrine disruption [68]. In this study, we found that the sex ratio of the offspring was decreased in a dose-dependent manner. *In utero* exposure is widely considered t the most sensitive exposure time in terms of reproductive effects [69]. Studies have shown that the mammalian hormone levels around the time of conception are associated with the sex of the resulting offspring [70]. Parental exposure to both dioxin and vinclozolin has been shown to cause excess female offspring [69], [71] due to altered hormone concentrations. In the present study, we found that the serum E_2_/T ratio was higher in the CYP groups, which may account for the decreased male-to-female sex ratio. In addition, the observed fetal death sites *in utero* after CYP treatment indicated that CYP affects male fetal development. Even we could not figure out the precise mechanism in this study, this decreased sex ratio resulting from CYP exposure should arouse the attention of researchers and policy makers. Does CYP affect the proteins that regulate fetal formation and maternal-fetus interface, or could it directly affect the genes controlling sex? The mechanism should be elucidated further.

Although the gene expression profiles were mostly similar between the *in vivo* and *in vitro* CYP treatment conditions, the gene expression of *ERα* differed between the two conditions. It is known that the localization of *ERα* is different during different testicular development stages [72]. In the fetal testis, *ERα* is present in Leydig cells only [73], whereas in the neonatal testis, *ERα* is present in Leydig cells, rete testis [74], and efferent ductules [74], [75]. In the adult testis, *ERα* expression is also found in round spermatids [76]. CYP may not influence *ERα* expression in regions other than Leydig cells, which may explain the difference in the results between the *in vitro* and the *in vivo* conditions.

In summary, our study determined that maternal low-dose CYP exposure during the perinatal stage impairs steroidogenesis and spermatogenesis in male offspring, which may have long-term effects on male fertility. These results have been found in mice, and our findings suggest that CYP may also impair testicular development in humans.

## Supporting Information

Figure S1 **Sex determination of offspring using through** ***Sry*** **gene analysis.** The sex of the offspring was determined through morphology and amplification of the *Sry* gene. F1–F8, offspring of one maternal mouse treated with high-dose CYP; a negative and positive control were also used. *L19* was used as an internal control.
(TIF)

Figure S2 **Concentration of CYP in the prepared dosages and the mother and offspring mouse sera.** The CYP concentrations of all of the samples were determined by ELISA. The concentrations of the prepared dosages were 20,243.5, 214,363.5, and 2,204,083.4 ppb, which are consistent with the expected values (23,980.81, 239,808.1 and 2,398,081 ppb). The serum concentrations of the CYP-treated maternal mice were 5.04, 34.82, and 169.8405 ppb, and those in the offspring ranged from 0.57 to 7.63 ppb (low dosage to high dosage). The residual concentrations in the sera of the mother and offspring mice in the vehicle group were close to zero. The data represent the mean ± SEM.
(TIF)

Figure S3 **Fetal death** ***in utero*** **of oil- and CYP-treated groups.** (A) Utero at E14.5 from oil-treated mouse. (B) Utero at E14.5 from high dose CYP-treated mouse, several fetal death sites (arrow) were observed in CYP-treated mouse. C, CYP.
(TIF)

Figure S4 **Time course expression of steroidogenesis genes at PD21.5, PD45.5, and PD90.5.** The mRNA levels of *Star*, *Cyp11a1*, *3β-HSD*, *Cyp17a1*, *17β-HSD3*, and *Cyp19a1* in the testes at PD21.5, PD45.5, and PD90.5 were measured using RT-PCR. All of the genes were significantly increased at PD45.5 and PD90.5 compared with the levels observed at PD21.5. The data represent the mean ± SEM. *indicates a significant difference between the group and the PD21.5 group; #indicates a significant difference between PD45.5 and PD90.5, * or ^#^ *P*<0.05.
(TIF)

Figure S5 **Effects of maternal perinatal CYP exposure on** ***Insl3*** **level of offspring.** The mRNA levels of *Insl3* in the different treatment groups at PD21.5, PD45.5, and PD90.5. CYP treatment can decrease the *Insl3* level at the three time points. The data represent the mean ± SEM. *indicates a significant difference between the group and control group, * P<0.05.
(TIF)

Figure S6 **Effects of CYP+LH treatment on steroidogenesis-related genes and** ***AR*** **,** ***Erα*** **, and** ***Pcna*** **levels in mLTC-1 cells.** (A) mRNA levels of steroidogenesis-related genes in mLTC-1 cells. (B) mRNA levels of mLTC-1 *AR*, *ERα*, and *Pcna*. (C) Media T levels of mLTC-1 cells. CYP+LH reduced the induction effect of LH on *Star*, *Cyp11a1*, *AR*, and *ERα*, but the mRNA levels were still higher than those observed in the vehicle group. *3β-HSD*, *cyp17a1*, and *17β-HSD3* were downregulated by CYP+LH treatment, whereas LH alone had a minor inhibitory effect on the expression of these genes. *Cyp19a1* and *Pcna* were upregulated by CYP+LH treatment, whereas LH alone increased the expression of these genes. The media T levels were decreased in the 10^−6^ and 10^−5^ M CYP+LH groups compared with the LH group. The data represent the mean ± SEM. *indicates a significant difference between the group and the control group, #indicates a significant difference between the group and LH group, *or^ #^ *P*<0.05. C, CYP.
(TIF)

## References

[1] Caserta D, Maranghi L, Mantovani A, Marci R, Maranghi F, et al (2008) Impact of endocrine disruptor chemicals in gynaecology. Hum Reprod Update 14: 59–72. 18070835 10.1093/humupd/dmm025

[2] Colborn T, vom Saal FS, Soto AM (1993) Developmental effects of endocrine-disrupting chemicals in wildlife and humans. Environ Health Perspect 101: 378–384. 8080506 10.1289/ehp.93101378 PMC1519860

[3] Kjeldsen LS, Ghisari M, Bonefeld-Jorgensen EC (2013) Currently used pesticides and their mixtures affect the function of sex hormone receptors and aromatase enzyme activity. Toxicol Appl Pharmacol. 10.1016/j.taap.2013.06.028 23871939

[4] Berteau Peter E, Knaak James B, Mengle Donald C, Schreider Jay B (1988) Insecticide Absorption from Indoor Surfaces. Biological Monitoring for Pesticide Exposure: American Chemical Society. pp. 315–326.

[5] Prendergast T PB, Jackson RJ, Slagel T, Kizer KW, Lyman DO (1989) Endrin poisoning associated with taquito ingestion—California. MMWR Morb Mortal Wkly Rep 38: 345–347. 2497330

[6] Russell HH, Jackson RJ, Spath DP, Book SA (1987) Chemical contamination of California drinking water. West J Med 147: 615–622. 3321714 PMC1025976

[7] Elbetieha A, Da'as SI, Khamas W, Darmani H (2001) Evaluation of the toxic potentials of cypermethrin pesticide on some reproductive and fertility parameters in the male rats. Arch Environ Contam Toxicol 41: 522–528. 11598791 10.1007/s002440010280

[8] Choi H, Moon JK, Liu KH, Park HW, Ihm YB, et al (2006) Risk assessment of human exposure to cypermethrin during treatment of mandarin fields. Arch Environ Contam Toxicol 50: 437–442. 16502205 10.1007/s00244-005-1050-3

[9] Jin Y, Wang L, Ruan M, Liu J, Yang Y, et al (2011) Cypermethrin exposure during puberty induces oxidative stress and endocrine disruption in male mice. Chemosphere 84: 124–130. 21397294 10.1016/j.chemosphere.2011.02.034

[10] Wang H, Wang SF, Ning H, Ji YL, Zhang C, et al (2011) Maternal cypermethrin exposure during lactation impairs testicular development and spermatogenesis in male mouse offspring. Environ Toxicol 26: 382–394. 20131380 10.1002/tox.20566

[11] Bhunya SP, Pati PC (1988) Genotoxic effects of a synthetic pyrethroid insecticide, cypermethrin, in mice in vivo. Toxicol Lett 41: 223–230. 3376150 10.1016/0378-4274(88)90058-6

[12] Rodriguez H, Tamayo C, Inostroza J, Soto C, Bustos-Obregon E, et al (2009) Cypermethrin effects on the adult mice seminal glands. Ecotoxicol Environ Saf 72: 658–662. 18490057 10.1016/j.ecoenv.2008.03.015

[13] Song L, Wang YB, Sun H, Yuan C, Hong X, et al (2008) Effects of fenvalerate and cypermethrin on rat sperm motility patterns in vitro as measured by computer-assisted sperm analysis. J Toxicol Environ Health A 71: 325–332. 18214806 10.1080/15287390701738517

[14] Wu W, Zhang J, Zhu W, Zheng YF, Zhu HJ, et al (2008) [Antiandrogenic effects of cypermethrin and beta-cypermethrin]. Zhonghua Lao Dong Wei Sheng Zhi Ye Bing Za Zhi 26: 193–197. 18724888

[15] Hu JX, Li YF, Li J, Pan C, He Z, et al (2013) Toxic effects of cypermethrin on the male reproductive system: with emphasis on the androgen receptor. J Appl Toxicol 33: 576–585. 22147539 10.1002/jat.1769

[16] Sun H, Xu XL, Xu LC, Song L, Hong X, et al (2007) Antiandrogenic activity of pyrethroid pesticides and their metabolite in reporter gene assay. Chemosphere 66: 474–479. 16857237 10.1016/j.chemosphere.2006.05.059

[17] Hu JX, Li YF, Pan C, Zhang JP, Wang HM, et al (2012) Anti-androgen effects of cypermethrin on the amino- and carboxyl-terminal interaction of the androgen receptor. Toxicology 292: 99–104. 22172556 10.1016/j.tox.2011.11.019

[18] Al-Hamdani NM, Yajurvedi HN (2010) Cypermethrin reversibly alters sperm count without altering fertility in mice. Ecotoxicol Environ Saf 73: 1092–1097. 20435348 10.1016/j.ecoenv.2010.04.009

[19] Bigsby R, Chapin RE, Daston GP, Davis BJ, Gorski J, et al (1999) Evaluating the effects of endocrine disruptors on endocrine function during development. Environ Health Perspect 107 Suppl 4613–618. 10.1289/ehp.99107s4613 PMC1567510 10421771

[20] Skakkebaek NE, Rajpert-De Meyts E, Main KM (2001) Testicular dysgenesis syndrome: an increasingly common developmental disorder with environmental aspects. Hum Reprod 16: 972–978. 11331648 10.1093/humrep/16.5.972

[21] Swan SH, Main KM, Liu F, Stewart SL, Kruse RL, et al (2005) Decrease in anogenital distance among male infants with prenatal phthalate exposure. Environ Health Perspect 113: 1056–1061. 16079079 10.1289/ehp.8100 PMC1280349

[22] Kjeldsen LS, Ghisari M, Bonefeld-Jorgensen EC (2013) Currently used pesticides and their mixtures affect the function of sex hormone receptors and aromatase enzyme activity. Toxicol Appl Pharmacol 272: 453–464. 23871939 10.1016/j.taap.2013.06.028

[23] Andersen HR, Schmidt IM, Grandjean P, Jensen TK, Budtz-Jorgensen E, et al (2008) Impaired reproductive development in sons of women occupationally exposed to pesticides during pregnancy. Environ Health Perspect 116: 566–572. 18414644 10.1289/ehp.10790 PMC2290975

[24] Carbone P, Giordano F, Nori F, Mantovani A, Taruscio D, et al (2007) The possible role of endocrine disrupting chemicals in the aetiology of cryptorchidism and hypospadias: a population-based case-control study in rural Sicily. Int J Androl 30: 3–13. 16824044 10.1111/j.1365-2605.2006.00703.x

[25] Komarek M, Cadkova E, Chrastny V, Bordas F, Bollinger JC (2010) Contamination of vineyard soils with fungicides: a review of environmental and toxicological aspects. Environ Int 36: 138–151. 19913914 10.1016/j.envint.2009.10.005

[26] Kristensen P, Irgens LM, Andersen A, Bye AS, Sundheim L (1997) Birth defects among offspring of Norwegian farmers, 1967–1991. Epidemiology 8: 537–544. 9270956 10.1097/00001648-199709000-00011

[27] Weidner IS, Moller H, Jensen TK, Skakkebaek NE (1998) Cryptorchidism and hypospadias in sons of gardeners and farmers. Environ Health Perspect 106: 793–796. 9831539 10.1289/ehp.98106793 PMC1533236

[28] Wohlfahrt-Veje C, Main KM, Schmidt IM, Boas M, Jensen TK, et al (2011) Lower birth weight and increased body fat at school age in children prenatally exposed to modern pesticides: a prospective study. Environ Health 10: 10–79. 21933378 10.1186/1476-069X-10-79 PMC3196902

[29] Kumari B, Madan VK, Kathpal TS (2008) Status of insecticide contamination of soil and water in Haryana, India. Environ Monit Assess 136: 239–244. 17406996 10.1007/s10661-007-9679-1

[30] Laabs V, Amelung W, Pinto AA, Wantzen M, da Silva CJ, et al (2002) Pesticides in surface water, sediment, and rainfall of the northeastern Pantanal basin, Brazil. J Environ Qual 31: 1636–1648. 12371181 10.2134/jeq2002.1636

[31] Jaensson A, Scott AP, Moore A, Kylin H, Olsen KH (2007) Effects of a pyrethroid pesticide on endocrine responses to female odours and reproductive behaviour in male parr of brown trout (Salmo trutta L.). Aquat Toxicol 81: 1–9. 17174415 10.1016/j.aquatox.2006.10.011

[32] LeBlanc L, Kuivila K (2008) Occurrence, distribution and transport of pesticides into the Salton Sea Basin, California, 2001–2002. In: Hurlbert S, editor. The Salton Sea Centennial Symposium: Springer Netherlands. pp. 151–172.

[33] Bedi JS, Gill JP, Aulakh RS, Kaur P, Sharma A, et al (2013) Pesticide residues in human breast milk: risk assessment for infants from Punjab, India. Sci Total Environ 463–464: 720–726. 10.1016/j.scitotenv.2013.06.066 23850662

[34] Berta P, Hawkins JR, Sinclair AH, Taylor A, Griffiths BL, et al (1990) Genetic evidence equating SRY and the testis-determining factor. Nature 348: 448–450. 2247149 10.1038/348448A0

[35] Wallis MC, Waters PD, Graves JA (2008) Sex determination in mammals—before and after the evolution of SRY. Cell Mol Life Sci 65: 3182–3195. 18581056 10.1007/s00018-008-8109-z PMC11131626

[36] Lin W, Rahman NA, Lin J, Zhang H, Gou K, et al (2011) Molecular mechanisms of bladder outlet obstruction in transgenic male mice overexpressing aromatase (Cyp19a1). Am J Pathol 178: 1233–1244. 21356374 10.1016/j.ajpath.2010.11.056 PMC3070572

[37] Lu D, Wang D, Feng C, Jin Y, Zhou Z, et al (2013) Urinary concentrations of metabolites of pyrethroid insecticides in textile workers, Eastern China. Environ Int 60: 137–144. 24056321 10.1016/j.envint.2013.08.004

[38] Wielgomas B, Nahorski W, Czarnowski W (2013) Urinary concentrations of pyrethroid metabolites in the convenience sample of an urban population of Northern Poland. Int J Hyg Environ Health 216: 295–300. 23021951 10.1016/j.ijheh.2012.09.001

[39] Barr DB, Olsson AO, Wong LY, Udunka S, Baker SE, et al (2010) Urinary concentrations of metabolites of pyrethroid insecticides in the general U.S. population: National Health and Nutrition Examination Survey 1999–2002. Environ Health Perspect 118: 742–748. 20129874 10.1289/ehp.0901275 PMC2898848

[40] Becker K, Seiwert M, Angerer J, Kolossa-Gehring M, Hoppe HW, et al (2006) GerES IV pilot study: assessment of the exposure of German children to organophosphorus and pyrethroid pesticides. Int J Hyg Environ Health 209: 221–233. 16461005 10.1016/j.ijheh.2005.12.002

[41] Ueyama J, Kimata A, Kamijima M, Hamajima N, Ito Y, et al (2009) Urinary excretion of 3-phenoxybenzoic acid in middle-aged and elderly general population of Japan. Environ Res 109: 175–180. 19081088 10.1016/j.envres.2008.09.006

[42] Khan DA, Hashmi I, Mahjabeen W, Naqvi TA (2010) Monitoring health implications of pesticide exposure in factory workers in Pakistan. Environ Monit Assess 168: 231–240. 19669582 10.1007/s10661-009-1107-2

[43] Hannas BR, Lambright CS, Furr J, Evans N, Foster PMD, et al (2012) Genomic Biomarkers of Phthalate-Induced Male Reproductive Developmental Toxicity: A Targeted RT-PCR Array Approach for Defining Relative Potency. Toxicological Sciences 125: 544–557. 22112501 10.1093/toxsci/kfr315 PMC3262859

[44] Ahmad M, Hussain I, Khan A, Najib ur R (2009) Deleterious effects of cypermethrin on semen characteristics and testes of dwarf goats (Capra hircus). Exp Toxicol Pathol 61: 339–346. 19019642 10.1016/j.etp.2008.10.002

[45] Holdcraft RW, Braun RE (2004) Hormonal regulation of spermatogenesis. Int J Androl 27: 335–342. 15595952 10.1111/j.1365-2605.2004.00502.x

[46] Roy AK, Lavrovsky Y, Song CS, Chen S, Jung MH, et al (1999) Regulation of androgen action. Vitam Horm 55: 309–352. 9949684 10.1016/s0083-6729(08)60938-3

[47] Mendis-Handagama SM, Ariyaratne HB (2001) Differentiation of the adult Leydig cell population in the postnatal testis. Biol Reprod 65: 660–671. 11514326 10.1095/biolreprod65.3.660

[48] Rey RA, Musse M, Venara M, Chemes HE (2009) Ontogeny of the androgen receptor expression in the fetal and postnatal testis: its relevance on Sertoli cell maturation and the onset of adult spermatogenesis. Microsc Res Tech 72: 787–795. 19551717 10.1002/jemt.20754

[49] Merlet J, Moreau E, Habert R, Racine C (2007) Development of fetal testicular cells in androgen receptor deficient mice. Cell Cycle 6: 2258–2262. 17890904 10.4161/cc.6.18.4654

[50] Li X, Nokkala E, Yan W, Streng T, Saarinen N, et al (2001) Altered structure and function of reproductive organs in transgenic male mice overexpressing human aromatase. Endocrinology 142: 2435–2442. 11356692 10.1210/endo.142.6.8211

[51] Li X, Rahman N (2008) Impact of androgen/estrogen ratio: lessons learned from the aromatase over-expression mice. Gen Comp Endocrinol 159: 1–9. 18762187 10.1016/j.ygcen.2008.07.025

[52] Hikim AP, Wang C, Leung A, Swerdloff RS (1995) Involvement of apoptosis in the induction of germ cell degeneration in adult rats after gonadotropin-releasing hormone antagonist treatment. Endocrinology 136: 2770–2775. 7750502 10.1210/endo.136.6.7750502

[53] Stephan H, Polzar B, Rauch F, Zanotti S, Ulke C, et al (1996) Distribution of deoxyribonuclease I (DNase I) and p53 in rat testis and their correlation with apoptosis. Histochem Cell Biol 106: 383–393. 8911966 10.1007/BF02473297

[54] El-Gohary M, Awara WM, Nassar S, Hawas S (1999) Deltamethrin-induced testicular apoptosis in rats: the protective effect of nitric oxide synthase inhibitor. Toxicology 132: 1–8. 10199576 10.1016/s0300-483x(98)00114-0

[55] Ma A, Yang X, Wang Z, Shi D, Chen Y (2008) Adult exposure to diethylstilbestrol induces spermatogenic cell apoptosis in vivo through increased oxidative stress in male hamster. Reprod Toxicol 25: 367–373. 18296022 10.1016/j.reprotox.2007.12.007

[56] Dohle GR, Smit M, Weber RF (2003) Androgens and male fertility. World J Urol 21: 341–345. 14566423 10.1007/s00345-003-0365-9

[57] Blanco-Rodriguez J, Martinez-Garcia C (1998) Apoptosis precedes detachment of germ cells from the seminiferous epithelium after hormone suppression by short-term oestradiol treatment of rats. Int J Androl 21: 109–115. 9675620 10.1046/j.1365-2605.1998.00109.x

[58] Krishnan AV, Stathis P, Permuth SF, Tokes L, Feldman D (1993) Bisphenol-A: an estrogenic substance is released from polycarbonate flasks during autoclaving. Endocrinology 132: 2279–2286. 8504731 10.1210/endo.132.6.8504731

[59] Zhang G-L, Zhang X-F, Feng Y-M, Li L, Huynh E, et al (2013) Exposure to bisphenol A results in a decline in mouse spermatogenesis. Reproduction, Fertility and Development 25: 847–859. 10.1071/RD12159 22951085

[60] Cheek AO, Vonier PM, Oberdorster E, Burow BC, McLachlan JA (1998) Environmental signaling: a biological context for endocrine disruption. Environ Health Perspect 106 Suppl 15–10. 9539003 10.1289/ehp.106-1533276 PMC1533276

[61] Lolicato F, Marino R, Paronetto MP, Pellegrini M, Dolci S, et al (2008) Potential role of Nanos3 in maintaining the undifferentiated spermatogonia population. Dev Biol 313: 725–738. 18089289 10.1016/j.ydbio.2007.11.011

[62] Symonds DA, Tomic D, Miller KP, Flaws JA (2005) Methoxychlor induces proliferation of the mouse ovarian surface epithelium. Toxicol Sci 83: 355–362. 15525693 10.1093/toxsci/kfi024

[63] Anderson EL, Baltus AE, Roepers-Gajadien HL, Hassold TJ, de Rooij DG, et al (2008) Stra8 and its inducer, retinoic acid, regulate meiotic initiation in both spermatogenesis and oogenesis in mice. Proc Natl Acad Sci U S A 105: 14976–14980. 18799751 10.1073/pnas.0807297105 PMC2542382

[64] Oulad-Abdelghani M, Bouillet P, Decimo D, Gansmuller A, Heyberger S, et al (1996) Characterization of a premeiotic germ cell-specific cytoplasmic protein encoded by Stra8, a novel retinoic acid-responsive gene. J Cell Biol 135: 469–477. 8896602 10.1083/jcb.135.2.469 PMC2121034

[65] Zhou Q, Nie R, Li Y, Friel P, Mitchell D, et al (2008) Expression of stimulated by retinoic acid gene 8 (Stra8) in spermatogenic cells induced by retinoic acid: an in vivo study in vitamin A-sufficient postnatal murine testes. Biol Reprod 79: 35–42. 18322276 10.1095/biolreprod.107.066795 PMC3208264

[66] Schrader M, Muller-Tidow C, Ravnik S, Muller M, Schulze W, et al (2002) Cyclin A1 and gametogenesis in fertile and infertile patients: a potential new molecular diagnostic marker. Hum Reprod 17: 2338–2343. 12202422 10.1093/humrep/17.9.2338

[67] Liu D, Matzuk MM, Sung WK, Guo Q, Wang P, et al (1998) Cyclin A1 is required for meiosis in the male mouse. Nat Genet 20: 377–380. 9843212 10.1038/3855

[68] James WH (2006) Offspring sex ratios at birth as markers of paternal endocrine disruption. Environmental Research 100: 77–85. 15922323 10.1016/j.envres.2005.03.001

[69] Mocarelli P, Gerthoux PM, Ferrari E, Patterson DG Jr, Kieszak SM, et al (2000) Paternal concentrations of dioxin and sex ratio of offspring. Lancet 355: 1858–1863. 10866441 10.1016/S0140-6736(00)02290-X

[70] James WH (2008) Evidence that mammalian sex ratios at birth are partially controlled by parental hormone levels around the time of conception. Journal of Endocrinology 198: 3–15. 18577567 10.1677/JOE-07-0446

[71] Zober A, Hoffmann G, Ott MG, Will W, Germann C, et al (1995) Study of morbidity of personnel with potential exposure to vinclozolin. Occup Environ Med 52: 233–241. 7795738 10.1136/oem.52.4.233 PMC1128201

[72] O'Donnell L, Robertson KM, Jones ME, Simpson ER (2001) Estrogen and spermatogenesis. Endocr Rev 22: 289–318. 11399746 10.1210/edrv.22.3.0431

[73] Greco TL, Furlow JD, Duello TM, Gorski J (1992) Immunodetection of estrogen receptors in fetal and neonatal male mouse reproductive tracts. Endocrinology 130: 421–429. 1727715 10.1210/endo.130.1.1727715

[74] Fisher JS, Millar MR, Majdic G, Saunders PT, Fraser HM, et al (1997) Immunolocalisation of oestrogen receptor-alpha within the testis and excurrent ducts of the rat and marmoset monkey from perinatal life to adulthood. J Endocrinol 153: 485–495. 9204003 10.1677/joe.0.1530485

[75] Nielsen M, Bjornsdottir S, Hoyer PE, Byskov AG (2000) Ontogeny of oestrogen receptor alpha in gonads and sex ducts of fetal and newborn mice. J Reprod Fertil 118: 195–204. 10793642 10.1530/jrf.0.1180195

[76] Pelletier G, Labrie C, Labrie F (2000) Localization of oestrogen receptor alpha, oestrogen receptor beta and androgen receptors in the rat reproductive organs. J Endocrinol 165: 359–370. 10810300 10.1677/joe.0.1650359

