# Supplementary figures and images for: Maternal Cypermethrin Exposure during the Perinatal Period Impairs Testicular Development in C57BL Male Offspring
Source: PLoS One. 2014 May 8;9(5):e96781. doi: 10.1371/journal.pone.0096781 (PMC4014553; doi:10.1371/journal.pone.0096781)

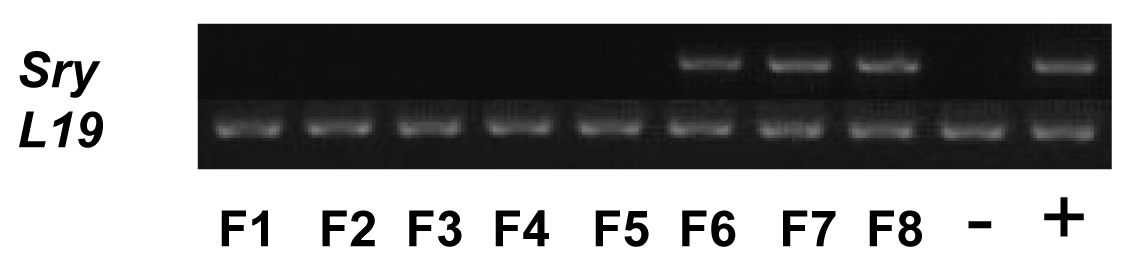

Supplement: Figure S1 — Sex determination of offspring using through Sry gene analysis. The sex of the offspring was determined through morphology and amplification of the Sry gene. F1–F8, offspring of one maternal mouse treated with high-dose CYP; a negative and positive control were also used. L19 was used as an internal control. (TIF) [file pone.0096781.s001.tif]

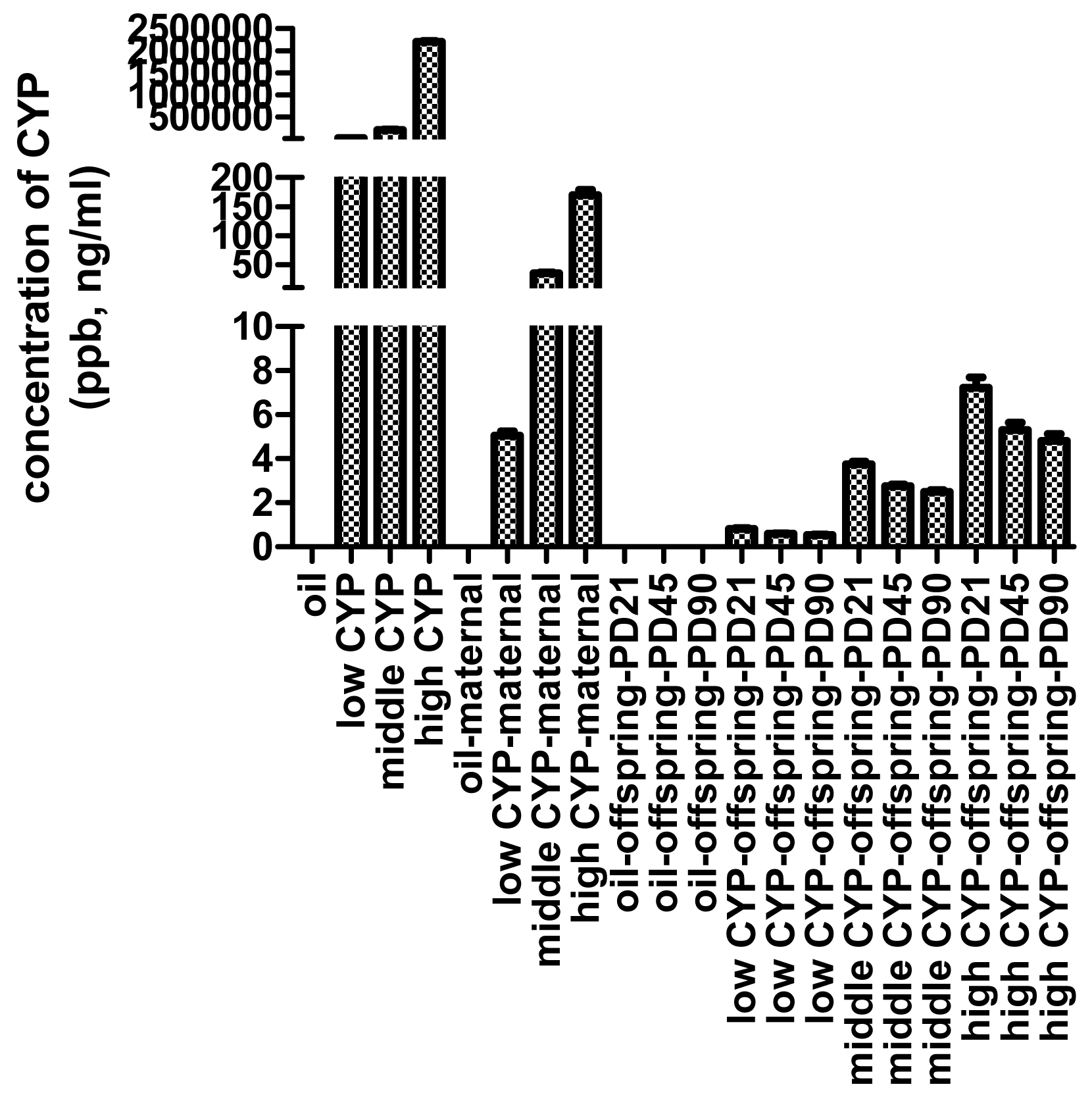

Supplement: Figure S2 — Concentration of CYP in the prepared dosages and the mother and offspring mouse sera. The CYP concentrations of all of the samples were determined by ELISA. The concentrations of the prepared dosages were 20,243.5, 214,363.5, and 2,204,083.4 ppb, which are consistent with the expected values (23,980.81, 239,808.1 and 2,398,081 ppb). The serum concentrations of the CYP-treated maternal mice were 5.04, 34.82, and 169.8405 ppb, and those in the offspring ranged from 0.57 to 7.63 ppb (low dosage to high dosage). The residual concentrations in the sera of the mother and offspring mice in the vehicle group were close to zero. The data represent the mean ± SEM. (TIF) [file pone.0096781.s002.tif]

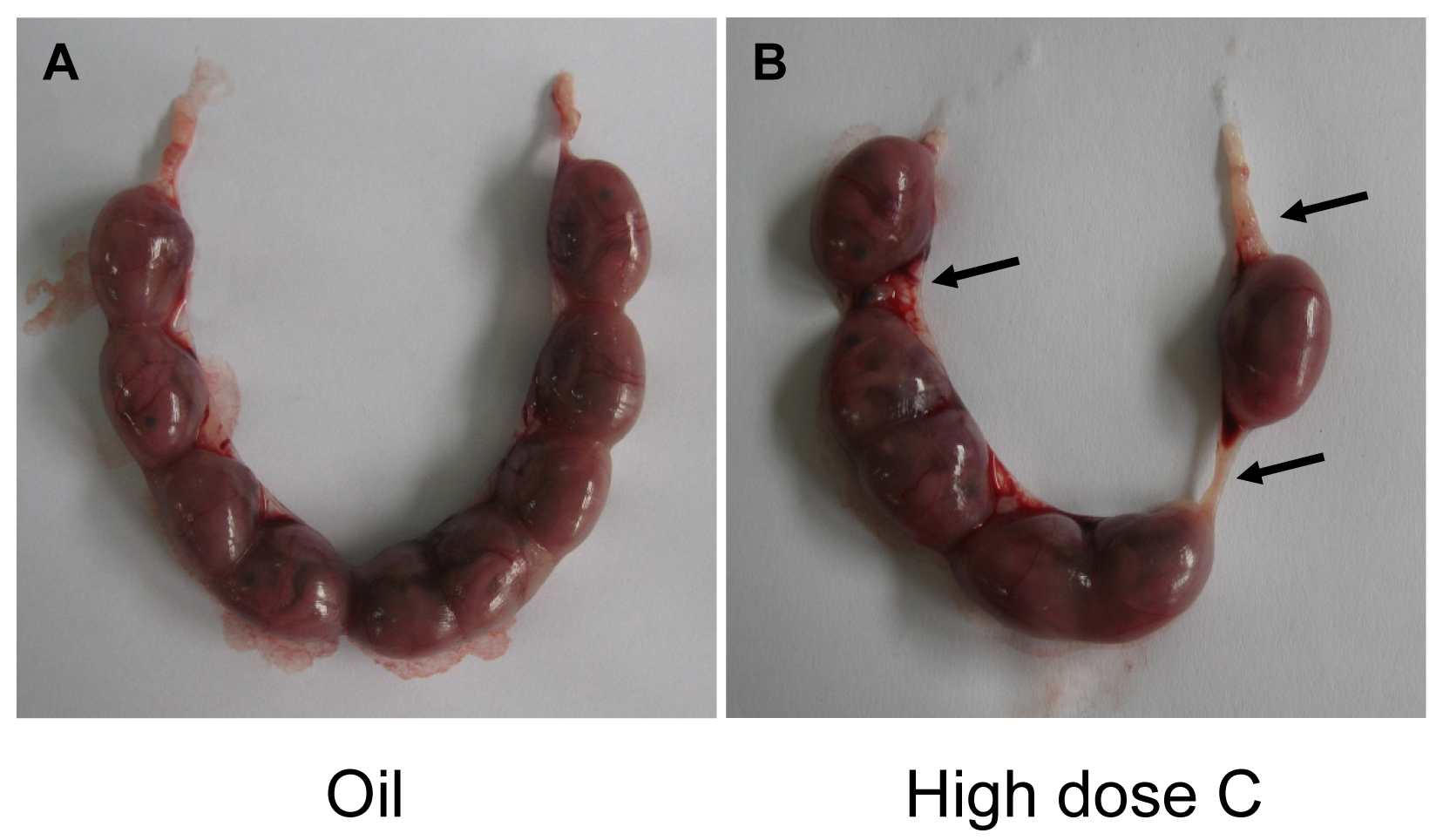

Supplement: Figure S3 — Fetal death in utero of oil- and CYP-treated groups. (A) Utero at E14.5 from oil-treated mouse. (B) Utero at E14.5 from high dose CYP-treated mouse, several fetal death sites (arrow) were observed in CYP-treated mouse. C, CYP. (TIF) [file pone.0096781.s003.tif]

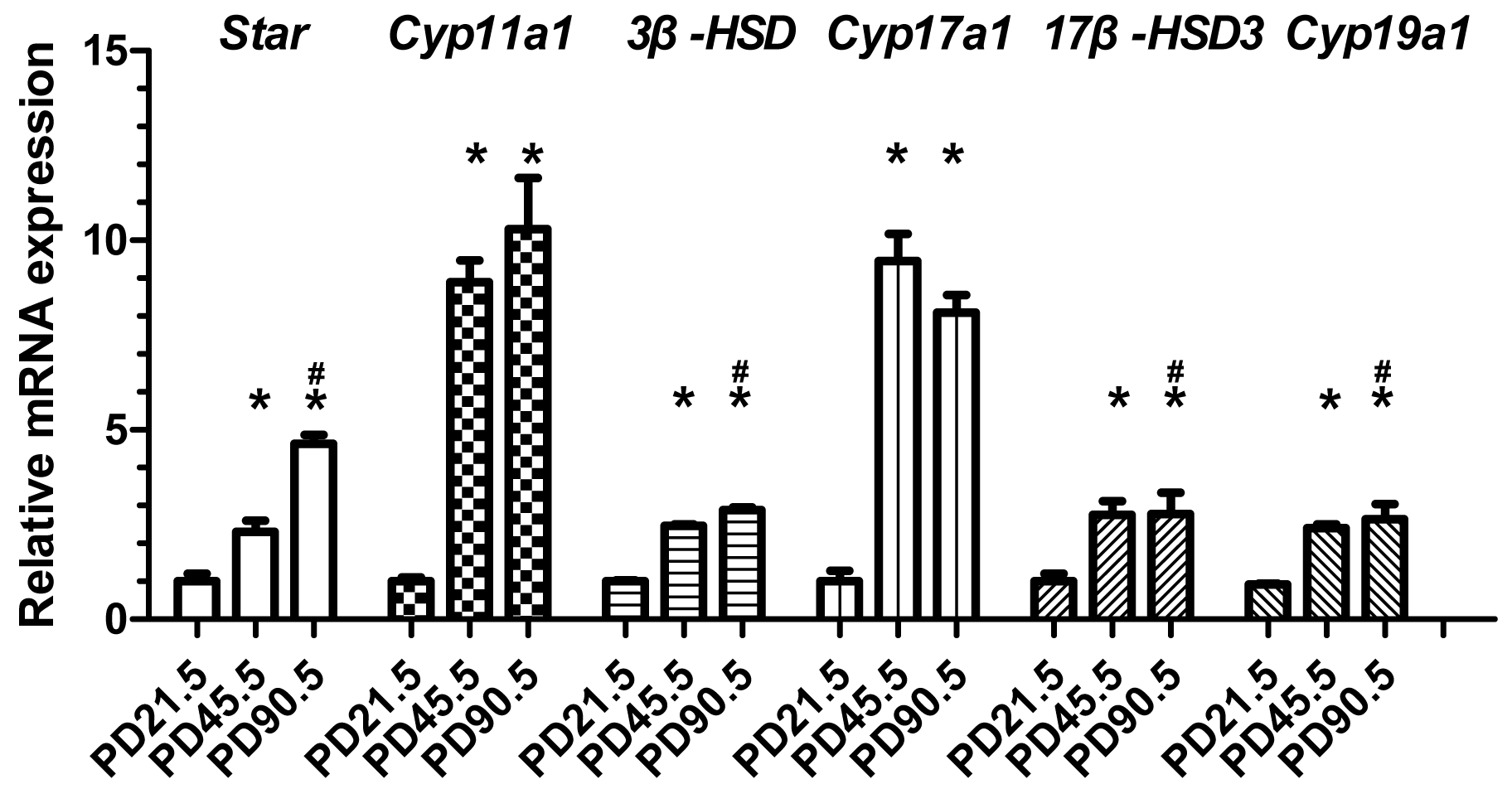

Supplement: Figure S4 — Time course expression of steroidogenesis genes at PD21.5, PD45.5, and PD90.5. The mRNA levels of Star, Cyp11a1, 3β-HSD, Cyp17a1, 17β-HSD3, and Cyp19a1 in the testes at PD21.5, PD45.5, and PD90.5 were measured using RT-PCR. All of the genes were significantly increased at PD45.5 and PD90.5 compared with the levels observed at PD21.5. The data represent the mean ± SEM. *indicates a significant difference between the group and the PD21.5 group; #indicates a significant difference between PD45.5 and PD90.5, * or # P<0.05. (TIF) [file pone.0096781.s004.tif]

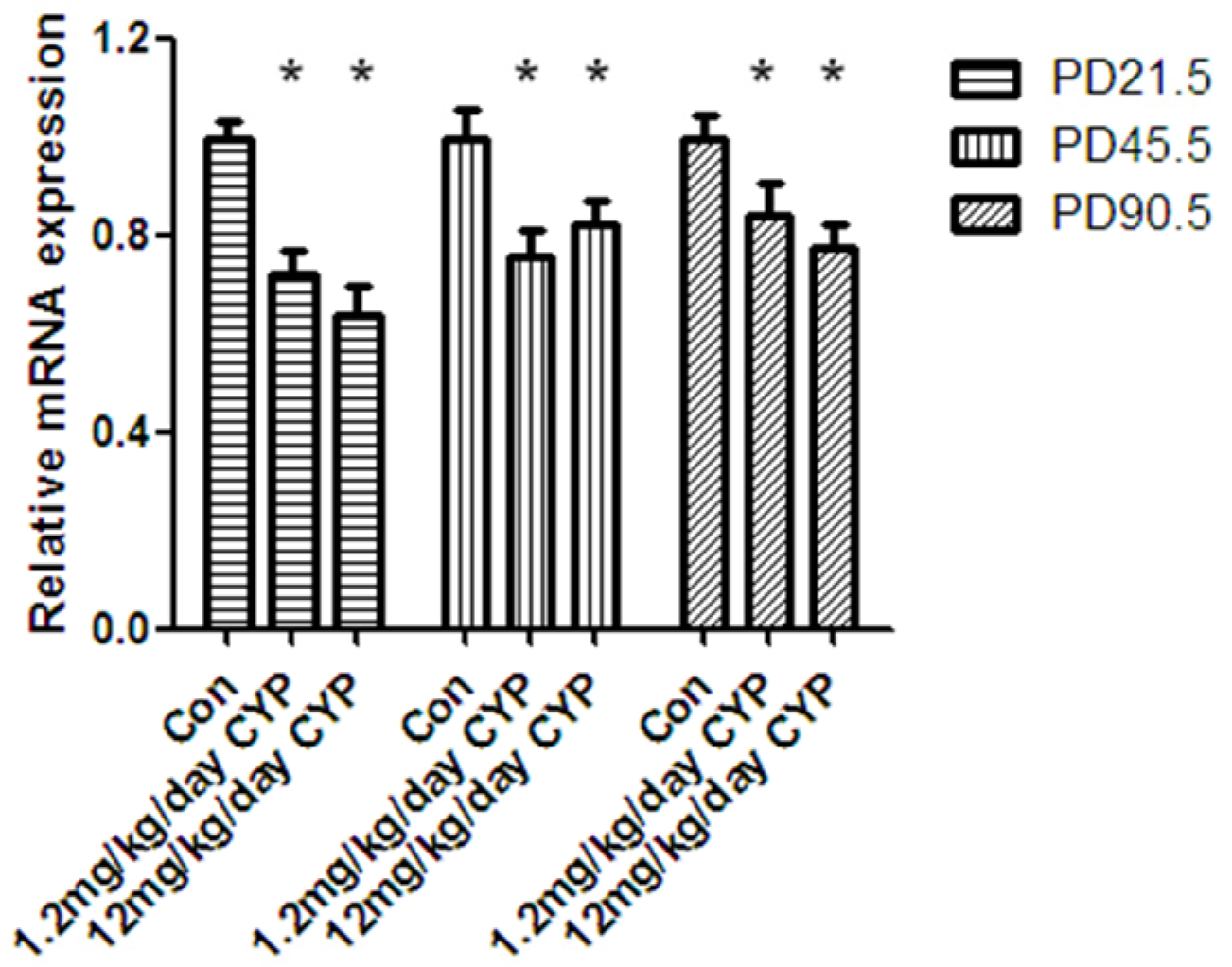

Supplement: Figure S5 — Effects of maternal perinatal CYP exposure on Insl3 level of offspring. The mRNA levels of Insl3 in the different treatment groups at PD21.5, PD45.5, and PD90.5. CYP treatment can decrease the Insl3 level at the three time points. The data represent the mean ± SEM. *indicates a significant difference between the group and control group, * P<0.05. (TIF) [file pone.0096781.s005.tif]

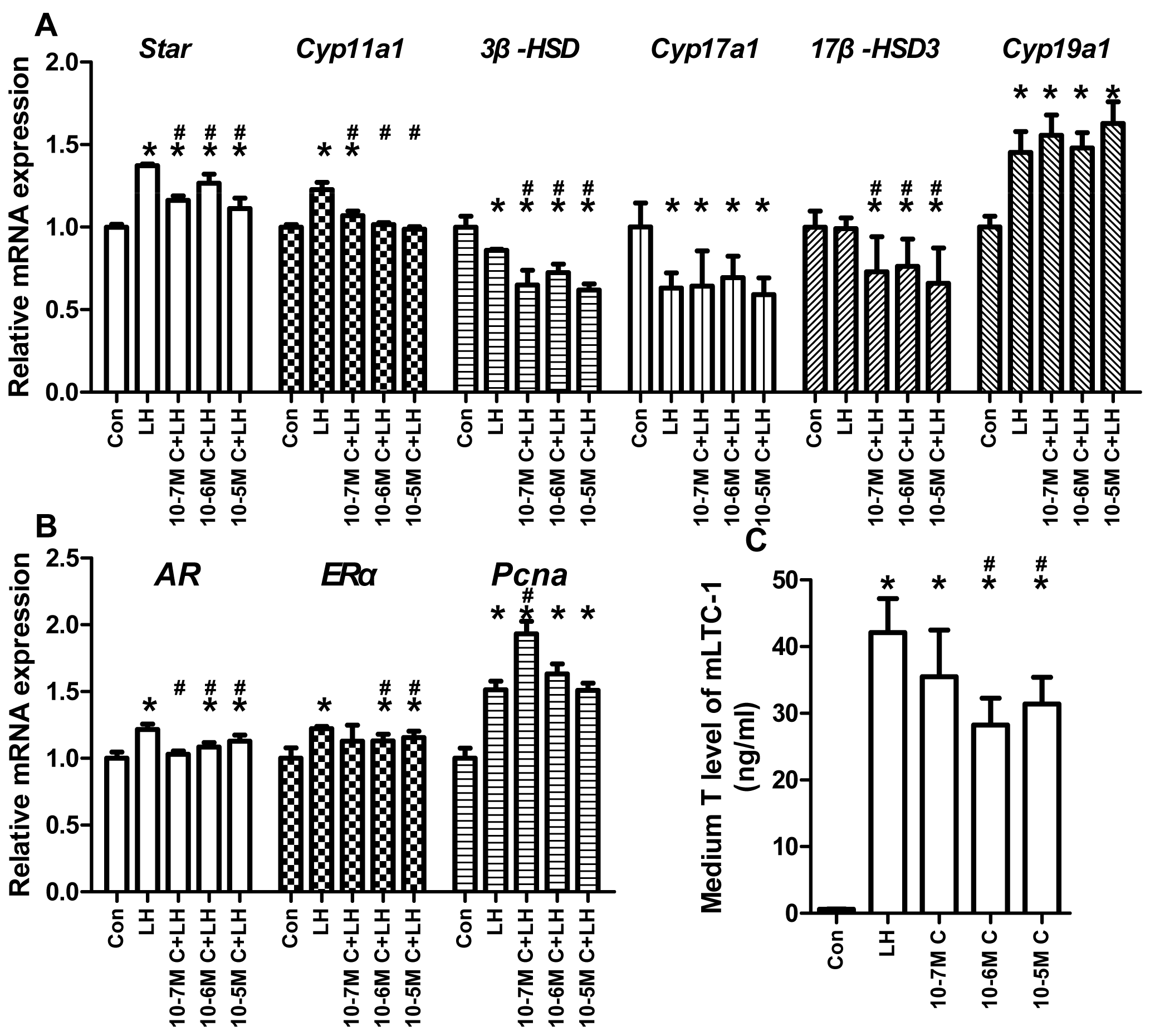

Supplement: Figure S6 — Effects of CYP+LH treatment on steroidogenesis-related genes and AR , Erα , and Pcna levels in mLTC-1 cells. (A) mRNA levels of steroidogenesis-related genes in mLTC-1 cells. (B) mRNA levels of mLTC-1 AR, ERα, and Pcna. (C) Media T levels of mLTC-1 cells. CYP+LH reduced the induction effect of LH on Star, Cyp11a1, AR, and ERα, but the mRNA levels were still higher than those observed in the vehicle group. 3β-HSD, cyp17a1, and 17β-HSD3 were downregulated by CYP+LH treatment, whereas LH alone had a minor inhibitory effect on the expression of these genes. Cyp19a1 and Pcna were upregulated by CYP+LH treatment, whereas LH alone increased the expression of these genes. The media T levels were decreased in the 10−6 and 10−5 M CYP+LH groups compared with the LH group. The data represent the mean ± SEM. *indicates a significant difference between the group and the control group, #indicates a significant difference between the group and LH group, *or # P<0.05. C, CYP. (TIF) [file pone.0096781.s006.tif]
